# Sex ratios at birth in Australia according to mother’s country of birth: A national study of all 5 614 847 reported live births 1997–2016

**DOI:** 10.1371/journal.pone.0251588

**Published:** 2021-06-25

**Authors:** Kristina Edvardsson, Mary-Ann Davey, Rhonda Powell, Anna Axmon

**Affiliations:** 1 Judith Lumley Centre, School of Nursing and Midwifery, La Trobe University, Bundoora Victoria, Australia; 2 Department of Obstetrics and Gynaecology, Monash University, Clayton, Victoria, Australia; 3 School of Nursing and Midwifery, La Trobe University, Bundoora Victoria, Australia; 4 Division of Occupational and Environmental Medicine, EPI@LUND (Epidemiology, Population studies, and Infrastructures at Lund University), Lund University, Lund, Sweden; University of New South Wales, AUSTRALIA

## Abstract

**Objectives:**

Son preference and sex selective practices have resulted in a deficit of girls in several countries, primarily across Asia. Emerging evidence indicates that son preference survives migration to Western high-income countries. The objective of this study was to assess male-to-female (M/F) ratios at birth per mother’s country of birth in Australia 1997–2016, in total and by parity, and by states/territories and over time.

**Methods:**

Data for this national population-based cross-sectional study were obtained from the National Perinatal Data Collection (NPDC) and included all live births in Australia 1997–2016 (N = 5 614 847). M/F ratios with 95% Confidence Intervals were estimated.

**Results:**

The M/F ratio for births to Australian-born mothers was within the expected range (1.03–1.07) regardless of parity and time period. M/F ratios were elevated above the expected range for births to mothers born in China in the total sample (M/F ratio 1.084, 95% confidence interval 1.071–1.097) and at parity 2 (1.175, 1.120–1.231), and for births to mothers born in India at parity 2 (1.146, 1.090–1.204). Parity 2 births were the most consistently male-biased across time. Across states, elevated M/F ratios were identified for both groups in New South Wales (China parity 2: 1.182, 1.108–1.260; India parity 2: 1.182, 1.088–1.285), for births to Chinese-born mothers in Victoria (total births: 1.097, 1.072–1.123; parity 1: 1.115, 1.072–1.159) and Australian Capital Territory (total births: 1.189, 1.085–1.302) and births to Indian-born mothers Western Australia (parity 2: 1.307, 1.122–1.523).

**Conclusions:**

Son preference persists in some immigrant communities after migration to Australia. The consistent pattern of elevated M/F ratios across the larger states indicates that sex imbalances at birth are largely independent of restrictiveness of local abortion laws. Drivers and consequences of son preference in Western high-income settings should be explored to further promote gender equality, and to strengthen support for women who may be vulnerable to reproductive coercion.

## Introduction

The practice of prenatal sex selection due to son preference has resulted in distortions of the sex ratio at birth in a number of countries across Asia [1], with China and India accounting for the largest proportion of ‘missing’ female births [1–3]. In the last 10 years, evidence of male-biased sex ratios in immigrant communities has also emerged in Western high-income countries, including Canada [4–8], Greece [9,10], Italy [11], Norway [12], Spain [13,14], Sweden [15], England and Wales [16], the USA [17–23] and one state in Australia [24], with some evidence of male-biased sex ratios persisting to the second generation [5,25] and among couples from different origins [7]. Consistent with patterns across Asia, sex ratios have been particularly biased at higher parities.

There are variations in the sex ratio from conception to adulthood, with a variety of determinants influencing the sex ratio across the life course [1,26]. While evidence regarding the sex ratio at conception is inconclusive [27], the natural or ‘expected’ sex ratio at birth is about 105 boys born for every 100 girls [1,28], a ratio that is fairly consistent across the globe [29] with only small fluctuations across regions, generally within the range of 104–106 [1]. It is also largely independent of birth order, the sex of the previous child or sex composition of siblings [30]. A sex ratio at birth above 107 is commonly seen as an indication of gender-biased sex selection [28]. Fetal sex can be determined through ultrasound, amniocentesis, chorionic villus sampling [31], and more recently, by a simple maternal blood test that can be performed from as early as 7 weeks of gestation [32]. According to the World Health Organization (WHO), prenatal sex selection most commonly occurs after conception through sex determination followed by abortion, but can also occur before conception through in vitro fertilisation (IVF) by pre-implantation sex identification and selection [33].

Sex selection through assisted reproduction is currently prohibited in Australia except in cases where it is medically indicated [34], however, there are media reports of Australian parents seeking selective IVF overseas to choose the sex of the baby [35]. In 2018, there were 14 355 infants born following assisted reproduction in Australia, which represents nearly one in 20 births [36]. Abortion legislation is inconsistent between Australian states and territories [37].

A summary is provided in S1 Table, Current Law for Non-Emergency Abortions in Australian States and Territories.

A 2018 study indicated male-biased sex ratios in the state of Victoria, Australia. The study, which was based on 1 191 250 births covering the period 1999–2015, revealed elevated male-to-female (M/F) ratios among births to mothers born in India, China, and South-East Asia, particularly at higher parities (e.g. in 2011–2015, 1.248 and 1.218 for China and India respectively at parity ≥ 2, and in 2005–2010 1.179 for South-East Asia at parity 1) [24]. Since no previous research has addressed the situation nationally in Australia, we aimed to assess M/F ratios at birth per mother’s country of birth for all live births in Australia 1997–2016, in total and by parity, using the expected range as point of reference; and to investigate whether any observed deviations were consistent across Australian states/territories and over time.

## Materials and methods

### Data source

Data for this cross-sectional study were obtained from the National Perinatal Data Collection (NPDC), a national population-based data collection of pregnancy and childbirth in Australia. NPDC includes data supplied for each birth by each state and territory and is compiled yearly by the Australian Institute of Health and Welfare (AIHW). Notification forms for each birth in Australia are completed by midwives or other birth attendants, and based on information from mothers, hospital or other records [38]. NPDC includes a Perinatal National Minimum Data Set (NMDS), an agreed set of perinatal variables for mandatory uniform supply by states and territories to support national reporting [39].

### Study population

Data on all live births and stillbirths were obtained for the period 1997–2016. Births included in the NPDC are defined as those at 20 or more weeks’ gestation or with a birth weight of at least 400g. Only live births were included in analyses because it was not otherwise possible to identify and exclude late terminations of pregnancy in all states and territories during the study period.

### Variables

#### Infant’s sex

Infants were categorised as female or male. 1 364 infants (<0.1%) were classified as ‘Indeterminate and not stated’ and were excluded from analyses. Individual case records allowed for analysis of each infant regardless of whether they were singleton or part of a multiple birth.

#### Mother’s country of birth

Country values for mother’s country of birth were provided according to the Australian Standard Classification of Countries for Social Statistics (ASCSS) 1990, the Standard Australian Classification of Countries (SACC) 1998, the Standard Australian Classification of Countries 2nd edition (SACC 2nd edition), the Standard Australian Classification of Countries 2011 (SACC2011) or pre-arranged groupings. Depending on the number of births, countries of birth were either analysed separately or collapsed into world regions, according to the SACC2011 [40]. In total 51 810 (<1%) country values for mothers could not be classified because of insufficient information.

#### Stratification factors

Births were categorised into parity (total number of previous pregnancies resulting in at least one live birth or stillbirth) 0, 1, 2 and ≥3, and infant’s birth state/territory: New South Wales, Victoria, Queensland, Western Australia, South Australia, Tasmania, Australian Capital Territory, and Northern Territory. The variables year of birth and state/territory were complete and 2.2% of cases had missing values for parity (n = 125 847). For Victoria, data for parity were not available for 2009 (n = 72 388). For Western Australia, prior to July 2014 the number of infants previously born was provided instead of parity, however, in analyses this variable was treated as parity.

### Statistical analyses and selection process

M/F ratios with 95% confidence intervals (CIs) were estimated using logistic regression with an intercept-only model. In addition to Australia, maternal countries of birth with at least 20 000 live births in Australia in the period 1997–2016 were analysed separately by country (all births combined as well as stratified by parity). The limit of 20 000 was set to allow for M/F ratios to be calculated with reasonable precision in stratified analyses [41]. Australia and other maternal countries of birth with at least one M/F ratio (i.e. either in the analysis of the total births or in analyses stratified by parity) with a CI entirely outside the range of 1.04–1.06 were selected for further analyses stratified by time period and state. All remaining countries in the dataset were collapsed into world regions according to the SACC2011 [40]. Based on a recent assessment of sex ratio at birth reference levels across countries and regions, we considered M/F ratios with CIs entirely outside 1.03–1.07 as statistically significantly different from the expected ratio [2]. Cases with missing sex or maternal country of birth were excluded from all analyses, and cases with missing parity were excluded from analyses stratified by parity. The numbers of births included in each analysis are presented in tables and figures. Results based on less than 100 observations are not presented. All analyses were performed using IBM SPSS Statistics version 25.

#### Selection process outcome

The initial selection process (i.e. countries of maternal birth with at least 20 000 live births) resulted in separate analysis of 16 countries. These were the UK (n = 171 158), New Zealand (n = 153 857), India (n = 112 807), China (n = 103 746), Vietnam (n = 86 516), the Philippines (n = 62 401), Lebanon (n = 42 656), South Africa (n = 32 336), Malaysia (n = 27 950), Indonesia (n = 27 710), Iraq (n = 26 418), the former Yugoslavia (n = 26 301), Sri Lanka (n = 25 003), Korea (n = 22 375), the USA (n = 20 998), and Fiji (n = 20 310).

Countries with M/F ratios outside the expected range and thus selected for further analyses were China (total births and parities 1 and 2) and India (parities 1 and 2). The remaining countries were collapsed into world regions. M/F ratios with 95% CIs by parity for countries with at least 20 000 births are displayed in S1 Fig, ‘Selection of maternal countries of birth for detailed analysis–M/F ratios by parity’.

### Ethical considerations

The study was approved by La Trobe University SHE College Human Ethics Sub-Committee (reference S17-135), the Australian Institute of Health and Welfare (AIHW) Ethics Committee (reference EO2018/1/428), the ACT Health Human Research Ethics Committee’s Low Risk Sub-Committee (reference 2018/LRE/00182), the Human Research Ethics Committee of the Northern Territory Department of Health and Menzies School of Health Research (HREC) (reference 2018–3224), Queensland Government, Department of Health, Health Innovation, Investment and Research Office (reference EO2018/1/428), Women’s and Children’s Health Network Human Research Ethics Committee, South Australia (reference HREC18/WCHN/78). Permission to extract data from the NPDC was also obtained from the Consultative Council on Obstetric and Paediatric Mortality and Morbidity, Victoria (reference 2018–09) and the Centre for Epidemiology, New South Wales Ministry of Health (reference H18/79234). The AIHW approval was sufficient for use of data from Western Australia and Tasmania, and so no further approvals were therefore sought from these states.

## Results

### Background characteristics

There were 5 614 847 live births reported in Australia between 1997 and 2016 of which 73.9% (n = 4 110 762) were to Australian-born mothers. The proportion of live births per state/territory were: New South Wales 32.7% (n = 1 836 662); Victoria 24.7% (n = 1 389 594); Queensland 20.0% (n = 1 121 938); Western Australia 10.4% (n = 583 161); South Australia 6.8% (n = 380 826); Tasmania 2.1% (n = 119 079); Australian Capital Territory 1.9% (n = 108 408); and Northern Territory 1.3% (n = 75 179).

Maternal, infant and birth-country characteristics are outlined in Table 1. On average mothers born in Australia, and in North Africa and the Middle East were the youngest (29.3 years), and mothers born in North East Asia were the oldest (32.5 years). The proportion of births per parity varied noticeably between countries and regions. Women born in India, and China and North East Asia had the lowest proportions of higher parity (2 and 3+) births.

**Table 1:** Maternal, infant and birth country characteristics of mothers giving birth in Australia 1997–2016.

Maternal characteristics							Infant characteristics				Birth country characteristics^a^	
Mother’s country of birth	N (% of births)	Mother’s age (years), mean (SD)	Parity no, (%)				Male sex (%)	Birth plurality, singleton (%)	Birthweight (grams), mean (SD)	Gestational age (weeks), mean (SD)	Total fertility	Male-to-female ratio at birth
			0	1	2	3+						
Australia	4 110 762 (73.9)	29.3 (5.6)	1 677 860 (41.7)	1 370 813 (34.1)	630 458 (15.7)	343 152 (8.5)	51.4	96.8	3 385 (590)	38.9 (2.1)	1.89	1.055^b^
China	103 746 (1.9)	31.5 (4.6)	55 540 (54.2)	38 533 (37.6)	6 951 (6.8)	1 355 (1.3)	52.0	98.0	3 318 (494)	38.9 (1.7)	1.60	1.16
India	112 807 (2.0)	29.7 (4.0)	62 246 (56.7)	40 026 (36.4)	6 283 (5.7)	1 305 (1.2)	51.6	97.8	3 147 (534)	38.7 (1.9)	2.44	1.11
Americas	71 867 (1.3)	32.0 (4.9)	33 460 (47.5)	24 194 (34.3)	8 899 (12.6)	3 931 (5.6)	51.1	97.0	3 393 (556)	38.9 (1.9)		
Europe	321 972 (5.8)	32.2 (5.0)	138 266 (43.7)	113 967 (36.0)	44 400 (14.0)	19 620 (6.2)	51.3	96.6	3 403 (567)	39.0 (1.9)		
North Africa and the Middle East	140 068 (2.5)	29.3 (5.6)	43 587 (32.4)	39 495 (29.4)	26 272 (19.5)	25 115 (18.7)	51.5	96.9	3 326 (550)	39.0 (2.0)		
North-East Asia (excl China)	64 664 (1.2)	32.5 (4.3)	34 292 (53.8)	22 460 (35.2)	5 769 (9.1)	1 216 (1.9)	51.6	97.8	3 242 (501)	38.9 (1.8)		
Oceania and Antarctica (excl Australia)	209 266 (3.8)	29.8 (5.8)	75 442 (37.3)	63 186 (31.2)	34 001 (16.8)	29 808 (14.7)	51.4	97.0	3 419 (602)	38.9 (2.0)		
Southern and Central Asia (excl India)	84 082 (1.5)	29.8 (4.8)	37 244 (45.6)	28 045 (34.3)	10 766 (13.2)	5 696 (7.0)	51.1	97.8	3 208 (548)	38.8 (1.9)		
South-East Asia	261 463 (4.7)	31.0 (5.1)	112 943 (44.1)	91 430 (35.7)	35 852 (14.0)	15 802 (6.2)	51.5	98.1	3 223 (516)	38.7 (1.9)		
Sub-Saharan Africa	82 340 (1.5)	30.9 (5.3)	30 425 (38.4)	26 780 (33.8)	12 812 (16.2)	9 218 (11.6)	51.2	96.8	3 326 (573)	38.9 (2.1)		

SD, standard deviation

^a^Birth country characteristics obtained from United Nations, Department of Economic and Social Affairs, Population Division (2017). World Population Prospects: The 2017 Revision, DVD Edition, data representing years 2010–2015.

^b^Data from current study, representing years 2010–2015.

Births to Indian-born mothers increased the most during the study period, from 1 481 births in 1997 to 14 507 in 2016, while births to Australian-born mothers ranged between 195 460 and 219 832 annually during the period, with the lowest and highest number of births in 2001 and 2007 respectively (S2 Fig ‘Number of births per year 1997–2016 by mother’s country or region of birth’).

### Male-to-female ratios overall and by parity

**Fig 1:**
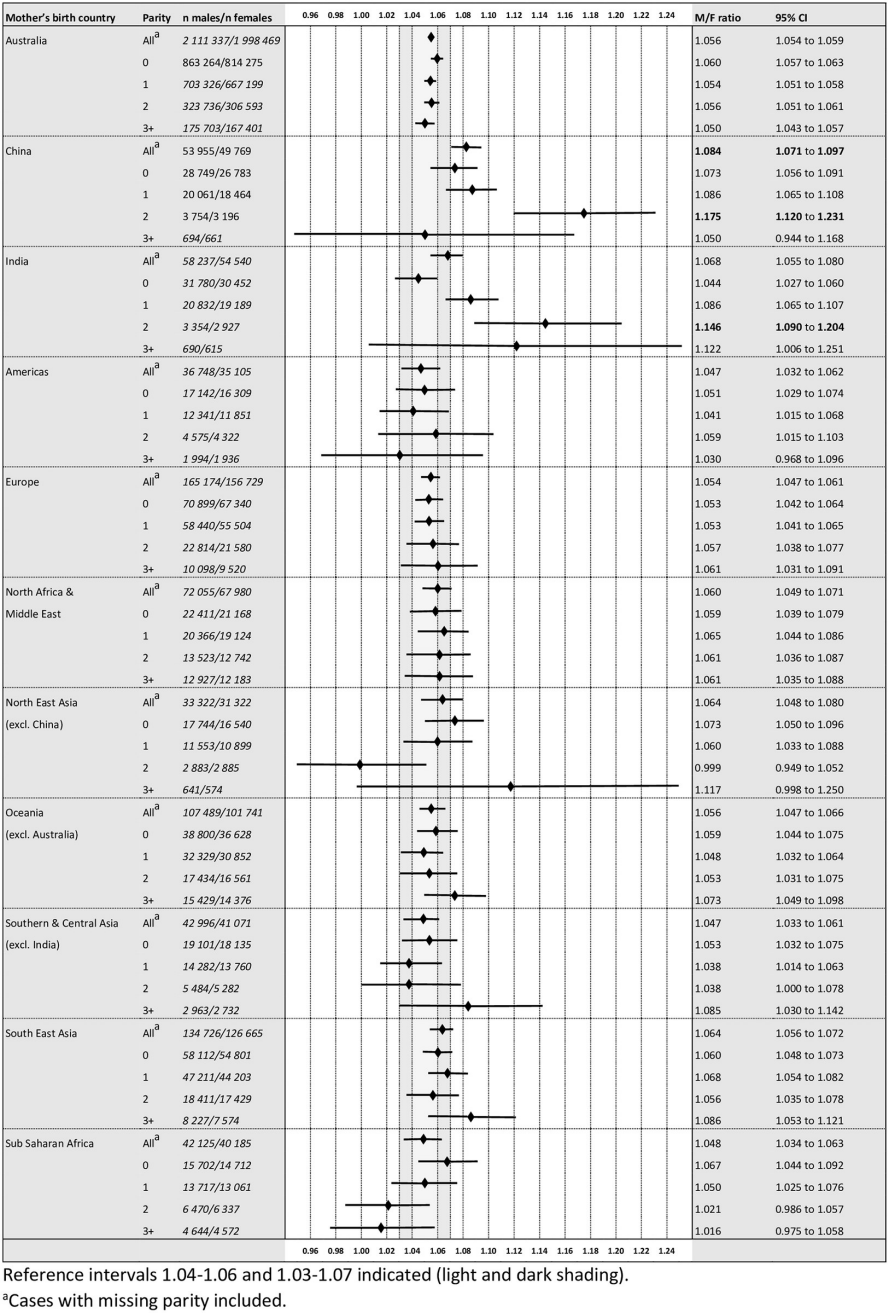
Male-to-female ratios stratified by parity.

The M/F ratio in Australia between 1997 and 2016 was 1.057 for all live births and 1.056 for births to Australian-born mothers. As shown in Fig 1, overall M/F ratios for all countries and regions ranged between 1.047 (Americas and Southern and Central Asia) and 1.084 (China), with China being statistically significantly elevated above the expected ratio.

M/F ratios for births to Australian-born mothers remained stable within the expected range, independent of parity, but were statistically significantly elevated for parity 2 births of Chinese and Indian-born mothers (1.175 and1.146 respectively). M/F ratios displayed a stepwise increase from parity 0 to 2 for both groups (Fig 1).

There was a trend towards increased point estimates for parity 3+ births in several regions, although none was statistically significant (Fig 1).

### Male-to-female ratios by parity and state/territory

The M/F ratios of births to Australian-born mothers fluctuated within the expected range in all states and territories, independent of parity.

M/F ratios for births to Chinese-born mothers were statistically significantly elevated above the expected range in New South Wales (parity 2: 1.182, CI 1.108–1.260, 2 033 males/1 720 females), Australian Capital Territory (total births: 1.189, CI 1.085–1.302, 1 008 males/848 females) and Victoria (total births: 1.097, CI 1.072–1.123, 14 920 males/13 599 females; parity 1: 1.115, CI 1.072–1.159, 5 424 males/4 866 females).

For births to Indian-born mothers, M/F ratios were statistically significantly elevated at parity 2 in New South Wales (1.182, CI 1.088–1.285, 1 212 males/1 025 females) and Western Australia (1.307, CI 1.122–1.523, 379 males/290 females). M/F ratios by state and parity are presented in S2 Table ‘Male-to-female ratios by maternal country of birth stratified by parity and state where infant was born’.

### Male-to-female ratio trends by parity

**Fig 2:**
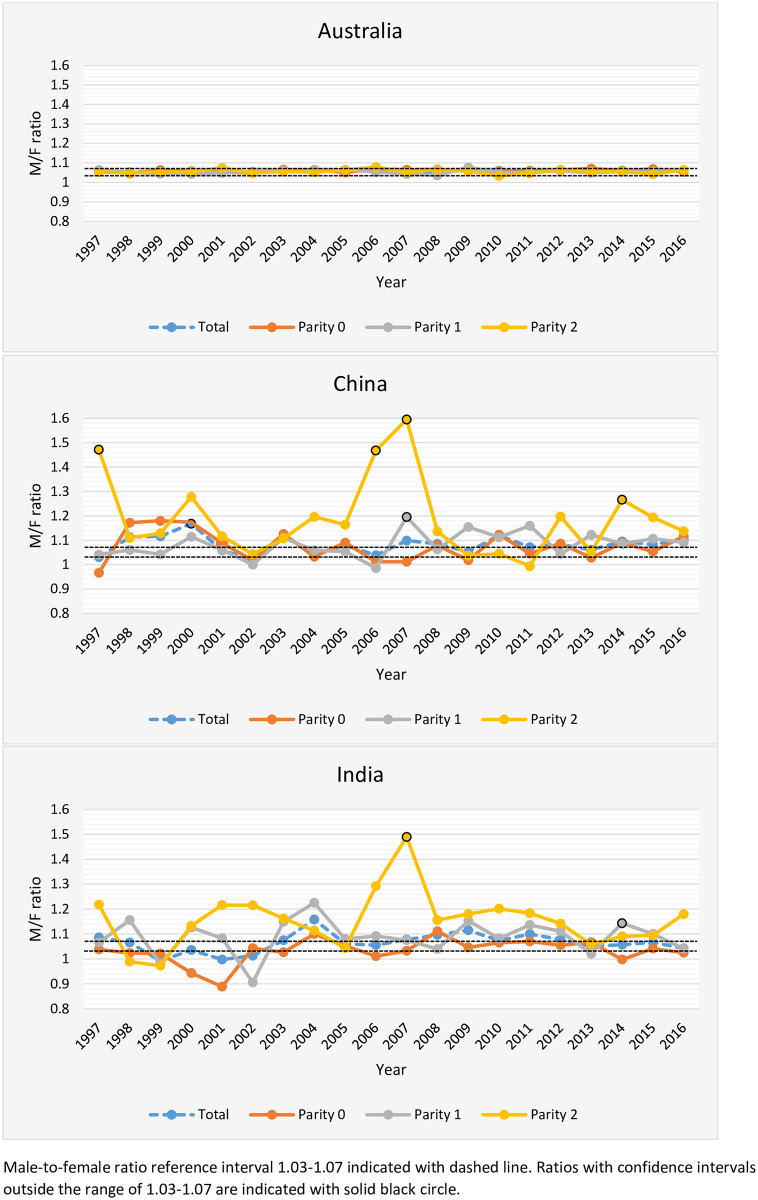
Male-to-female ratio trends by parity 1997–2016.

Point estimates for M/F ratios by year and parity are displayed in Fig 2. For births to Australian-born mothers the M/F ratios remained steady within the expected range independent of parity over the period 1997–2016.

For births to Chinese-born mothers, point estimates for parity 2-births were elevated above the expected range at the majority of time points (15/20 years), with statistically significant values observed in 1997 (1.471, CI 1.144–1.891) 2006 (1.468, CI 1.154–1.869) 2007 (1.595, CI 1.264–2.012) and 2014 (1.226, CI 1.076–1.490). Point estimates for parity 1 births were elevated at half of the time points, with a statistically significant value observed in 2007 (1.195, CI 1.074–1.331). The highest M/F ratio for total births was observed in year 2000 (1.168, CI 1.093–1.248), when point estimates were elevated above the expected range in all parity categories.

Similarly, for Indian-born mothers, parity 2-births had the most consistently elevated M/F ratio point estimates (16/20 years), with the highest and statistically significant value observed in 2007 (1.489, CI 1.143–1.939). M/F ratios for parity 1 fluctuated throughout the period, with most point estimates above the expected range and with a statistically significant deviation in 2014 (1.143, CI 1.080–1.211).

## Discussion

In this national population-based study of all live births in Australia 1997 to 2016, we found male-biased sex ratios for births to Chinese-born and Indian-born women, particularly at higher parities. The observed sex imbalances appeared largely consistent across time for both groups of women who had two previous births, and also across the largest states New South Wales and Victoria. Elevated ratios were also found in Australian Capital Territory, and in Western Australia. These findings confirm sex imbalances at birth across Australia among infants of mothers who have migrated from countries where son preference is documented and overall sex ratios at birth distorted in favour of males. These results are consistent with evidence from other Western high-income countries, where elevated M/F ratios have been documented among infants of mothers of Indian [4–9,11–14,16–21,23] and Chinese [9,11,13,17–21,23] origin.

This study includes all reported live births in Australia 1997–2016 (N = 5 614 847) which eliminates the risk of sampling and response bias. Australia’s perinatal reporting system is considered to be virtually complete. The large sample gave sufficient statistical power to enable analyses stratified by two factors simultaneously (i.e. combinations of parity, state/territory and time period) in most cases, with only a few exceptions. Estimates of M/F ratios were more precise in more recent time periods because of the increase in births by Chinese and Indian-born mothers over the study period, for the latter group nearly tenfold. Estimates were less precise and thus less meaningful in states with fewer births (e.g. Tasmania, Australian Capital Territory and Northern Territory represented 2.1%, 1.9% and 1.3% of all births in Australia, respectively).

The initial analyses of maternal countries of birth with at least 20 000 live births in Australia indicate point estimates of M/F ratios both below and above the expected range for countries other than China and India. However, none of these were statistically different from the expected ratio and therefore not analysed in further detail. The ‘expected’ sex ratio used as a reference interval for statistical significance (1.03–1.07) was based on previous research [2]. However, it is important to note that the sex ratio reference level is not fixed or known. While the natural or ‘expected’ M/F ratio is often quoted to be close to 1.05 [28], in their systematic assessment of sex ratios at birth across all countries, Chao et al (2019) estimated national reference levels to range from 1.031 to 1.067. According to Tafuro and Guilmoto (2020), variations in the sex ratio at birth are modest in countries with reliable statistics, which is indicated also in Australian data where over the 20-year period the national M/F ratios were stable. The subgroups analysed in this study only constitute a small proportion of Australian births, so the observed deviations are unlikely to influence national statistics.

Limitations of this study include missing parity in the national dataset for Victorian births in 2009, which affected analyses stratified by parity but not total sample analyses. Only live births were included which means the true sex ratio may be underestimated due to the higher stillbirth rate among boys [42]. It is possible that M/F ratio imbalances exist in other overseas-born sub-groups of the Australian population where the number of births is too small for statistical significance to be established. Furthermore, we were unable to identify the gender composition of older siblings, an influential factor for sex-selective choices. Previous studies from other Western high-income countries consistently show higher M/F ratios if the previous births were female, compared to if previous births were male or of mixed sex [5,8,13,15,19–21].

According to the United Nations Population Fund (UNFPA), a male-biased sex ratio is a cause for concern since it reflects the persistent low status of females [43]. A greater value placed on sons over daughters is linked to both cultural and economic factors and sets of social norms such as patrilineal inheritance, reliance on sons to provide economic and old age security and support, responsibility for death rituals and for continuation of the family line, while females in some settings may pose a burden to the family due to the dowry system [31,43,44]. UNFPA suggests that son preference and sex selection are linked but not synonymous, since there are parts of the world where son preference exists but where sex selective practices are uncommon [45]. The occurrence of prenatal sex selection is suggested to be a ‘product’ of three key factors, which in addition to son preference includes availability of prenatal diagnostic technology (for sex determination) and low fertility [1]. The implementation of strict family planning policies in some Asian countries (e.g. China) has contributed to the decline in fertility in these settings [1,45]. In our sample, the proportion of higher parity births was very low among Indian and Chinese-born mothers, 5.7% and 6.8% respectively at parity 2 (compared to 15.7% to Australian-born mothers), which indicates that families may be small following migration. This factor may thus reinforce the effect of son preference on sex ratios, since having a small family also means a lower chance of spontaneously having a son [46]. Globally, there are countries that previously have had sex ratio imbalances but where sex ratios now are at near expected levels (e.g. Republic of Korea, Singapore) or have levelled off (Vietnam), while China and India together are estimated to account for 90–95% of the world’s missing female births annually [45].

In this study there were some states where M/F ratios were elevated for births to either the Chinese-born or Indian-born group but not the other. These variations in M/F ratios between and within states may possibly be linked to socio-economic, religious [5] or cultural differences within groups of Chinese- and Indian-born women who settle in different regions of Australia. This is supported by UNFPA reports that there are large variations in sex ratios within countries affected by skewed sex ratios, with differences between regions, by wealth, educational level and family size [45]. In India, selective abortion of female pregnancies is suggested to be more common in richer households due to better access to sex determination and abortion services [47], however as affordability of sex selection technology increases, access is also suggested to be increasing for low-income families [45].

It is important to further explore links between son preference and women’s reproductive autonomy. A recent study from Australia on service providers’ views on reproductive coercion and abuse against women from minority ethnic backgrounds described that fetal sex could act as a ‘catalyst’ for reproductive coercion and abuse in the context of son preference. One example raised by providers was a woman revealing abuse from her partner and in-laws, which escalated when she was asked to but refused to terminate a female pregnancy [48]. Similar findings have been described in the United States, where a majority of 65 Indian immigrant women who had pursued sex selective abortions described their reproductive decision making as mediated by others, particularly the husband and mother-in-law. Several of the interviewed women had experienced physical abuse and neglect as a consequence of not having a son, including a partner’s attempt to forcibly terminate a (female) pregnancy [49]. These qualitative findings highlight the need for providing further context to the male-biased sex ratios observed in this study, including exploration of the divers and consequences of son preference in the migration context. Such evidence can also inform and strengthen the way health professionals support women who may be vulnerable to reproductive coercion and abuse in this context.

The most plausible pathway for the male-biased sex ratios observed in this study is through termination of pregnancy following sex determination. This pathway is likely due to the window that exists between the gestational age at which sex can be determined through common methods including Noninvasive Prenatal Testing, NIPT (from 7–10 weeks) [32,50] or ultrasound (from 12 weeks) [51], and the upper boundary for the gestational age at which termination can be performed upon request (selected states, see S1 Table). In the United States, Indian immigrant women who intended to pursue sex selection and were not given information about fetal sex before 20–22 weeks in standard prenatal care, turned to private clinics where this information could be offered from 12 weeks [49]. It seems likely that this pathway exists also in Australia. Furthermore, it may be impossible for health providers to identify situations where sex selection is the primary reason for seeking an abortion [45]. The role ultrasound plays in sex selection is well known, and the rise in sex ratios in selected countries have been linked to the increasing availability of ultrasound and other prenatal diagnostic techniques from the 1970s [1,2]. Concerns have been raised that the more recently introduced NIPT test, which only requires a simple maternal blood sample, may have the potential to facilitate pregnancy terminations due to unwanted fetal sex, but evidence is still only anecdotal [52]. The pathway through selective implantation is less likely in Australia, since sex selection through assisted reproduction is prohibited (unless medically indicated) [34], and the relatively low prevalence of assisted reproduction (about 1 in 20 infants born).

It could be anticipated that sex ratios would be more even in states where termination was not available on request (e.g. New South Wales prior to 2019). However, our results show elevated M/F ratios across a number of states, despite considerable differences in abortion legislation, which indicates that liberal abortion legislation did not result in greater rates of selective abortion of females. The two largest states New South Wales and Victoria account for 57% of births and, during the period of study, had very different abortion legislation and yet M/F ratios were elevated for parity 2 births to Chinese-born and Indian-born mothers in both states at nearly identical levels. Time trends indicate a peak in M/F ratios for parity 2-births for both groups in 2007, which was one year prior to the decriminalisation of termination in Victoria in 2008. Thus, although women may travel interstate for terminations [53,54], increased access to termination does not seem to have inflated sex ratio imbalances.

There is a lack of reliable data on how abortion laws are applied in practice in the various states, particularly given the extent of medical discretion to determine whether statutory criteria are met in some states, and it is not necessarily the case that sex selective abortion is harder to obtain in a state with conservative legislation. It is important to note also that women may travel overseas for either abortion or selective implantation though IVF. Thus, it remains unknown whether possible sex selection procedures occurred within the state of residence, within other parts of Australia or overseas, or what proportion of the observed gender imbalances are a result of selective implantation though IVF or abortions, or by other means. According to the UNFPA laws and policies have not shown to be effective in preventing sex selective abortions, rather, changes in social norms are suggested to be the key [45], something which is important to consider in any preventative effort.

## Conclusions

Our findings strengthen existing evidence on son preference in the context of migration to Western high-income countries, and make an important contribution to the existing evidence base by indicating that patterns of male-biased sex ratios at birth are largely independent of restrictiveness of local abortion laws. There is a pressing need to explore factors driving and influencing son preference and prenatal sex selection in migrant communities in Australia and other Western high-income countries, since avenues for prevention in these contexts are lacking. This includes decision-making processes, and consequences for individuals, families and society, and their implications for women’s reproductive and overall health. This knowledge could facilitate the development of culturally appropriate ways, and other structural and service system interventions, to prevent male-biased sex selection, to promote gender equality and to strengthen support for women who may be vulnerable to reproductive coercion. Attention must be given also to the broader issue of gender inequality, taking into account the rich cultural diversity of Australian society.

## Supporting information

S1 Fig Selection of maternal countries of birth for detailed analysis—Male-to-female ratios by parity. (DOCX)

S2 Fig Number of births per year 1997–2016 by mother’s country or region of birth. (DOCX)

S1 Table Current law for non-emergency abortions in Australian states and territories. (DOCX)

S2 Table Male-to-female ratios by maternal country of birth stratified by parity and state where infant was born. (DOCX)
