# Supplementary material for: Sex ratios at birth in Australia according to mother’s country of birth: A national study of all 5 614 847 reported live births 1997–2016
Source: PLoS One. 2021 Jun 25;16(6):e0251588. doi: 10.1371/journal.pone.0251588 (PMC8232452; doi:10.1371/journal.pone.0251588)
Supplement: S1 Fig — (DOCX) [file pone.0251588.s001.docx]

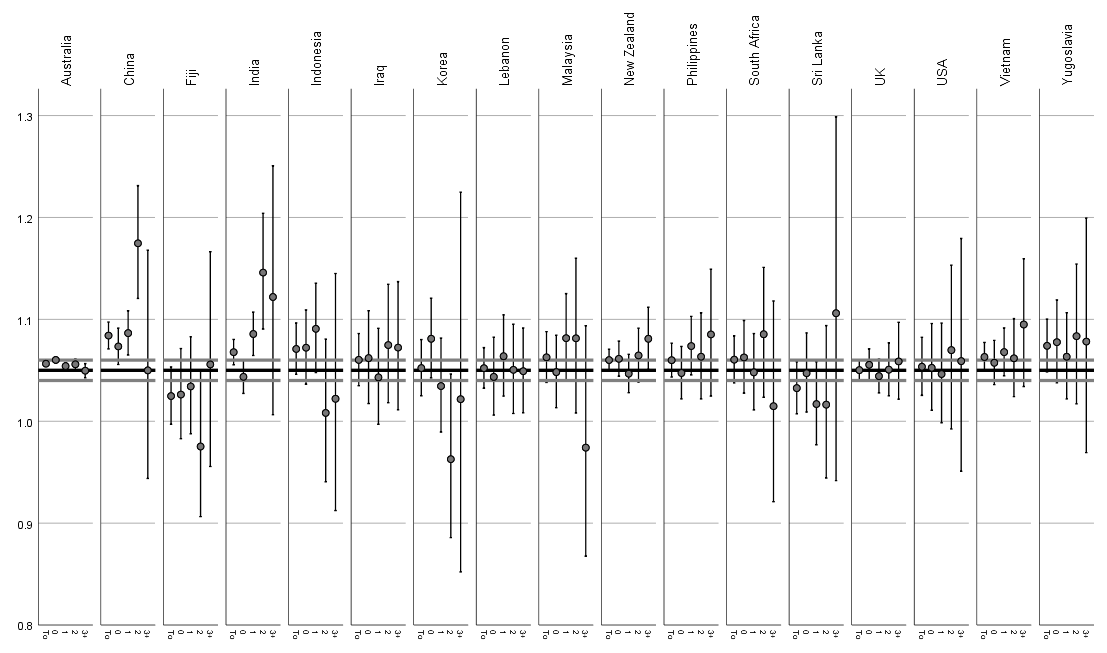


Parity

Male-to-female ratio at birth

**S2 Figure.** Selection of maternal countries of birth for detailed analysis – male-to-female ratios by parity*. Australia (n=4 109 806), UK (n=171 158), New Zealand (n=153 857), India (n=112 807), China (n=103 746), Vietnam (n=86 516), the Philippines (n=62 401), Lebanon (n=42 656), South Africa (n=32 336), Malaysia (n=27 950), Indonesia (n=27 710), Iraq (n=26 418), the former Yugoslavia (n=26 301), Sri Lanka (n=25 003), Korea (n=22 375), the USA (n=20 998), Fiji (n=20 310).

*Parity: To=all parities combined; 0=no prior birth; 1=1 prior birth; 2=2 prior births; 3+ = 3 or more prior births.
