# Supplementary material for: Sex ratios at birth in Australia according to mother’s country of birth: A national study of all 5 614 847 reported live births 1997–2016
Source: PLoS One. 2021 Jun 25;16(6):e0251588. doi: 10.1371/journal.pone.0251588 (PMC8232452; doi:10.1371/journal.pone.0251588)
Supplement: S2 Fig — (DOCX) [file pone.0251588.s002.docx]

**S3 Figure.** Number of births per year 1997-2016 by mother’s country or region of birth
