# Supplementary material for: Sex ratios at birth in Australia according to mother’s country of birth: A national study of all 5 614 847 reported live births 1997–2016
Source: PLoS One. 2021 Jun 25;16(6):e0251588. doi: 10.1371/journal.pone.0251588 (PMC8232452; doi:10.1371/journal.pone.0251588)
Supplement: S1 Table — (DOCX) [file pone.0251588.s003.docx]

| **State/ territory** | **Current law** | **Previous law** |
| --- | --- | --- |
| **New South Wales** | ***Abortion Law Reform Act 2019****:* On request to 22 weeks. After 22 weeks, lawful at an approved facility if 2 specialist doctors consider that there are sufficient grounds for a termination to be performed in all the circumstances (medical, current and future physical, psychological and social, and relevant professional standards and guidelines). | Until 2019: unlawful abortion a crime. *R v Wald* (1971) 2 DCR (NSW) 25: not unlawful “where there existed any economic, social, or medical ground or reason” upon which a doctor could base “an honest belief that [continuing the pregnancy] would result a serious danger to her physical or mental health.” |
| **Victoria** | ***Abortion Law Reform Act 2008****:* On request to 24 weeks. After 24 weeks, lawful if 2 doctors reasonably believe it appropriate in the circumstances (medical, current and future physical, psychological and social). | Until 2008: unlawful abortion a crime. *R v Davidson* (1969) VR 667 (SC) not unlawful if “necessary to preserve the woman from a serious danger to her life or physical or mental health … and in the circumstances not out of proportion to the danger to be averted.” |
| **Queensland** | ***Termination of Pregnancy Act 2018****:* On request to 22 weeks. After 22 weeks, lawful if 2 doctors agree it is necessary in the circumstances (medical, current and future physical, social and psychological, and relevant professional standards and guidelines). | Until 2018: only lawful to preserve a woman’s life or for the woman’s or unborn child’s benefit, if reasonable given the woman’s state at the time and all the circumstances. |
| **Western Australia** | [***Health Act 1911, ss 334-335***](http://www.austlii.edu.au/au/legis/wa/consol_act/ha191169/) (amended in 1998**)***:* Legal to 20 weeks if the woman will suffer serious personal, family or social consequences or serious danger to the physical or mental health. Very restricted after 20 weeks. | Prior to 1998: unlawful abortion a crime. Assumed that Queensland test for lawfulness applied. |
| **South Australia** | [***Criminal Law Consolidation Act 1935, s82A***](https://www.legislation.sa.gov.au/LZ/C/A/CRIMINAL%20LAW%20CONSOLIDATION%20ACT%201935.aspx)*:* Since 1969: lawful if 2 doctors form the opinion in good faith that continuation of pregnancy greater risk to physical or mental health of the woman than if terminated, or for serious fetal abnormality. Residency criteria: 2 months in South Australia. | N/A |
| **Tasmania** | ***Reproductive Health (Access to Terminations) Act 2013****:* On request to 16 weeks. Lawful after 16 weeks if 2 doctors agree that continuation of pregnancy greater risk to physical or mental health of the woman than if terminated, considering physical, social and psychological circumstances. No abortion services in Tasmania. | From 2001-2013, legal if 2 doctors form the opinion in good faith that continuation of pregnancy greater risk to physical or mental health of the woman than if terminated, or if serious fetal abnormality. Until 2001: Unlawful abortion a crime. Assumed that New South Wales or Victorian common law test for lawfulness applied. |
| **Australian Capital Territory** | ***Health Act 1993 (ACT) ss 80-84****:* All abortions are lawful if carried out by a doctor in an approved facility, provided it is prior to viability. | Until 2002: Unlawful abortion a crime. Common law test for lawfulness from *R v Wald (*NSW) applied. |
| **Northern Territory** | ***Termination of Pregnancy (Law Reform) Act 2017****:* Legal to 14 weeks with 1 doctor's approval, and at 14-23 weeks with 2 doctors’ approval. Doctor can approve if it is appropriate in the circumstances (medical, current and future physical, psychological and social circumstances, and professional guidelines). | Prior to 2017: unlawful abortion a crime. Lawful up to 14 weeks if continuation of pregnancy greater risk to physical or mental health of the woman than if terminated, or if serious fetal abnormality. |
