# Supplementary material for: Sex ratios at birth in Australia according to mother’s country of birth: A national study of all 5 614 847 reported live births 1997–2016
Source: PLoS One. 2021 Jun 25;16(6):e0251588. doi: 10.1371/journal.pone.0251588 (PMC8232452; doi:10.1371/journal.pone.0251588)
Supplement: S2 Table — (DOCX) [file pone.0251588.s004.docx]

| **Maternal country of birth** |  | **Parity** | | | |
| --- | --- | --- | --- | --- | --- |
| State/territory | **Total births^1^** | **0** | **1** | **2** | **3+** |
|  | M/F ratio (95% CI), *males/females* | M/F ratio (95% CI), *males/females* | M/F ratio (95% CI), *males/females* | M/F ratio (95% CI), *males/females* | M/F ratio (95% CI), *males/females* |
| **Australia** |  |  |  |  |  |
| New South Wales | 1.058 (1.054-1.061)  *652 897/617 371* | 1.064 (1.058-1.070)  *27 0292/25 4044* | 1.055 (1.049-1.061)  *21 8805/20 7403* | 1.053 (1.044-1.062)  *102 857/97 667* | 1.042 (1.030-1.055)  *55 389/53 131* |
| Victoria | 1.054 (1.050-1.058)  *508 494/482 514* | 1.058 (1.051-1.064) *205 693/19 4477* | 1.052 (1.044-1.059)  *167 162/158 954* | 1.050 (1.039-1.061)  *72 081/68 675* | 1.058 (1.041-1.074)  *32 568/30 788* |
| Queensland | 1.060 (1.055-1.064)  *460 945/435 054* | 1.061 (1.054-1.068)  *18 5003/17 4362* | 1.061 (1.053-1.069)  *152 006/143 257* | 1.056 (1.045-1.067)  *73 470/69 587* | 1.055 (1.041-1.068)  *45 962/43 585* |
| Western Australia | 1.049 (1.043-1.056)  *199 330/189 935* | 1.053 (1.043-1.064)  *82 721/78 532* | 1.043 (1.031-1.054)  *66 843/64 111* | 1.063 (1.046-1.080)  *30 930/29 110* | 1.038 (1.016-1.060)  *17 064/16 443* |
| South Australia | 1.053 (1.046-1.061)  *158 215/150 236* | 1.048 (1.037-1.060)  *65 501/62 474* | 1.051 (1.039-1.064)  55 631/52 910 | 1.063 (1.044-1.083)  *24 043/22 617* | 1.061 (1.034-1.089)  *12 097/12 097* |
| Tasmania | 1.075 (1.062-1.088)  *56 9908/53 022* | 1.082 (1.062-1.103)  *22 780/21 045* | 1.071 (1.050-1.093)  *19 158/17 885* | 1.072 (1.040-1.104) *9 140/8 530* | 1.059 (1.019-1.100)  *5 466/5 162* |
| Australian Capital Territory | 1.062 (1.047-1.076)  *42 890/40 393* | 1.069 (1.047-1.091)  *18 792/17 586* | 1.055 (1.031-1.080)  *14 891/14 114* | 1.069 (1.032-1.108)  *6 175/5 775* | 1.047 (0.993-1.103)  *2 847/2 720* |
| Northern Territory | 1.055 (1.038-1.071)  *31 576/29 944* | 1.062 (1.035-1.089)  *12 482/11 755* | 1.031 (1.001-1.062)  *8 830/8 565* | 1.088 (1.046-1.132)  *5 040/4 632* | 1.033 (0.990-1.078)  *4 310/4 173* |
| **China** |  |  |  |  |  |
| New South Wales | 1.073 (1.055-1.091)  *27 945/26 048* | 1.064 (1.040-1.089)  *15 014/14 109* | 1.067 (1.038-1.097)  *10 455/9 797* | **1.182 (1.108-1.260)**  *2 033/1720* | 1.041 (0.906-1.196)  *406/390* |
| Victoria | **1.097 (1.072-1.123)**  *14 920/13 599* | *1.078 (1.044-1.113)*  *7 784/7 223* | **1.115 (1.072-1.159)**  *5 424/4 866* | 1.174 (1.066-1.293)  *891/759* | 1.291 (1.024-1.628)  *891/759* |
| Queensland | 1.067 (1.023-1.113)  *4 454/4 174* | 1.058 (0.999-1.120)  *2 430/2 297* | 1.082 (1.009-1.160)  *1 648/1 523* | 1.137 (0.972-1.331)  *331/291* | 0.721 (0.490-1.063)  *44/61* |
| Western Australia | 1.103 (1.047-1.163)  *2 935/2 660* | 1.117 (1.037-1.203)  *1 489/1 333* | 1.081 (0.994-1.175)  *1 152/1 066* | 1.134 (0.947-1.357)  *254/224* | NA |
| South Australia | 1.113 (1.049-1.181)  *2318/2082* | 1.101 (1.017-1.192*)*  *1 275/1 158* | 1.140 (1.034-1.256)  *872/765* | 1.213 (0.955-1.542*)*  *148/122* | NA |
| Tasmania | 1.027 (0.854-1.235)  *228/222* | 1.129 (0.887-1.438)  *140/124* | 0.878 (0.640-1.205)  *72/82* | NA | NA |
| Australian Capital Territory | **1.189 (1.085-1.302**)  *1008/848* | 1.166 (1.032-1.318)  *554/475* | 1.192 (1.027-1.384)  *378/317* | 1.396 (0.964-2.022)  *67/48* | NA |
| Northern Territory | 1.081 (0.856-1.365*)*  *147/136* | 0.984 (0.695-1.394)  *63/64* | 1.250 (0.855-1.827)  *60/48* | NA | NA |
| **India** |  |  |  |  |  |
| New South Wales | 1.065 (1.044-1.087)  *19 648/18 443* | 1.035 (1.007-1.063)  10 683/10 325 | 1.089 (1.054-1.125)  7 479/6 868 | **1.182 (1.088-1.285)**  *1 212/1 025* | 1.217 (1.013-1.463)  *252/207* |
| Victoria | 1.070 (1.050-1.091)  *22 070/20 622* | 1.044 (1.017-1.071)  11 752/11 256 | 1.085 (1.050-1.122)  7 390/6 808 | 1.156 (1.062-1.258)  *1 157/1 001* | 0.991 (0.826-1.190)  *229/231* |
| Queensland | 1.054 (1.015-1.093)  *5 765/5 471* | 1.031 (0.983-1.081)  *3 433/3 330* | 1.040 (0.963-1.123)  *1 933/1 789* | 1.080 (1.013-1.152)  *318/287* | 1.317 (0.941-1.842)  *79/60* |
| Western Australia | 1.087 (1.045-1.131)  *5 174/4 759* | 1.048 (0.994-1.106)  *2 792/2 663* | 1.100 (1.031-1.174)  *1 901/1 728* | **1.307 (1.122-1.523)**  *379/290* | 1.282 (0.940-1.748)  *91/71* |
| South Australia | 1.079 (1.029-1.131)  *3 612/3 348* | 1.115 (1.047-1.186)  *2 081/1 867* | 1.040 (0.963-1.123)  *1 328/1 277* | 0.962 (0.781-1.183)  *175/182* | NA |
| Tasmania | 0.972 (0.802-1.177)  *205/211* | 0.863 (0.661-1.127)  *101/117* | 1.225 (0.896-1.676)  *87/71* | NA | NA |
| Australian Capital Territory | 1.016 (0.941-1.097)  *1 322/1 301* | 1.022 (0.920-1.134)  *708/693* | 1.065 (0.944-1.203)  *537/504* | 0.756 (0.551-1.035)  *68/90* | NA |
| Northern Territory | 1.145 (0.999-1.313)  *441/385* | 1.144 (0.947-1.383)  *230/201* | 1.229 (0.986-1.532)  *177/144* | NA | NA |
| ^1^Cases with missing parity included. M/F ratio = male-to-female ratio. CI = confidence interval. M/F ratios with CIs outside the range of 1.03-1.07 indicated in bold. NA: Total number of births less than 100. | | | | | |
